# Impact of the stress ulcer prophylactic protocol on reducing the unnecessary administration of stress ulcer medications and gastrointestinal bleeding: a single-center, retrospective pre-post study

**DOI:** 10.1186/s40560-020-0427-8

**Published:** 2020-01-16

**Authors:** Osamu Ogasawara, Taiki Kojima, Mitsunori Miyazu, Kazuya Sobue

**Affiliations:** 1 Department of Anesthesiology and Intensive Care Medicine, Nagoya City University Graduate School of Medical Science, 1-Kawasumi, Mizuho-cho, Mizuho-ku, Nagoya, Aichi 467-8601 Japan; 2 Department of Anesthesiology, Aichi Children’s Health and Medical Center, 7-426, Morioka-cho, Obu, Aichi 474-0031 Japan

**Keywords:** Hematemesis, Gastrointestinal hemorrhage, Melena, Critical care, Checklist

## Abstract

**Background:**

Clinically significant gastrointestinal bleeding from stress ulcers increases patient mortality in intensive care, and histamine type 2 receptor blockers and proton pump inhibitors as stress ulcer prophylaxes were reported to decrease the incidence of that.

Although medical checklists are widely used to maintain high compliance with medications and interventions to improve patient outcome in the intensive care field, the efficacy of medical checklists regarding the incidence of gastrointestinal bleeding and the reduction of unnecessary administration of stress ulcer prophylaxis medications has not been sufficiently explored to date.

This study aimed to investigate the incidence of gastrointestinal bleeding and the rate of administering stress ulcer prophylaxis medication before and after setting administration criteria for stress ulcer prophylaxis and introducing a medical checklist for critically ill adults.

**Methods:**

This was a retrospective pre-post study at a single-center, tertiary adult and pediatric mixed ICU. Adult patients (≥ 18 years) who were admitted to the ICU for reasons other than gastrectomy, esophagectomy, pancreatoduodenectomy, and gastrointestinal bleeding were analyzed. A medical checklist and stress ulcer prophylaxis criteria were introduced on December 22, 2014, and the patients were classified into the preintervention group (from September to December 21, 2014) and the postintervention group (from December 22, 2014, to April 2015). The primary outcome was the incidence of upper gastrointestinal bleeding, and the secondary outcome was the proportion administered stress ulcer prophylaxis medications.

**Results:**

One hundred adult patients were analyzed. The incidence of upper gastrointestinal bleeding in the pre- and postintervention groups was both 4.0% [95% confidence interval, 0.5–13.7%]. The proportion administered stress ulcer prophylaxis medications decreased from 100 to 38% between the pre- and post-intervention groups.

**Conclusions:**

After the checklist and the criteria were introduced, the administration of stress ulcer prophylaxis medications decreased without an increase in upper gastrointestinal bleeding in critically ill adults. Prospective studies are necessary to evaluate the causal relationship between the introduction of them and gastrointestinal adverse events in critically ill adults.

## Background

Stress ulcer prophylaxis (SUP) is an important concern in intensive care. Some previous studies have reported that 5 to 25% of critically ill patients without SUP develop upper gastrointestinal bleeding (UGIB) due to the severity of illness and/or invasive treatments [1, 2]. A systematic review and meta-analysis showed that histamine type 2 receptor blockers (H2Bs) and proton pump inhibitors (PPIs) significantly reduced the incidence of UGIB in critically ill adult patients [3]. Nonetheless, H2Bs and PPIs have been shown to increase the incidence of hospital-acquired pneumonia (HAP) and *Clostridioides difficile* infection (CDI) [4–6].

Currently, medical checklists are widely used to maintain the compliance of medications and interventions in intensive care units. Previous studies have reported that utilizing medical checklists improved the quality of patient care and reduced patient mortality and length of ICU stay [7–9].

However, the efficacy of medical checklists in terms of reducing the UGIB incidence and unnecessary administration of SUP medications has not been sufficiently explored yet.

This study aimed to investigate the incidence of UGIB and rate of administering SUP medications before and after setting administration criteria for SUP and introducing a medical checklist in critically ill adults.

## Methods

### Study design

This was a retrospective pre-post intervention, noninferiority study conducted at a single-center, tertiary adult and pediatric mixed ICU. Institutional ethical committee approval was obtained (approval number 60-19-0014). In this study, we analyzed patients admitted to the ICU between September 2014 and April 2015. At our ICU, we used a routine set of orders that included maintenance fluid and H2B together to recall the administration of H2B as a SUP that potentially resulted in the unnecessary administration of H2B as SUP in the preintervention period. If there was a contraindication for H2B, we administered PPI instead of H2B.

### Intervention

The interventions comprised the following three actions: introduction of a medical checklist (Table 1), removal of H2B from the routine set of orders that included maintenance fluid and H2B together, and creating SUP criteria (Table 2). These interventions were introduced on December 22, 2014. Prior to introducing these interventions, all the ICU medical professionals were instructed about the medical checklist and SUP criteria through department conferences and didactic lectures. The SUP criteria were created based on the reported criteria in previous reports [11–13].

**Table 1:** Checklist used in the ICU

Contents	Assessment
Analgesia	□Good □Bad
Sedation	□Good □Bad
Delirium	□Yes □No □Cannot evaluate (RASS, − 4 to − 5)
Medical restraint	□Applied □Necessary □Not necessary
Mechanical ventilation	
Spontaneous mode or SBT	□Applied □Possible □Impossible
Airway extubation	□Possible □Impossible
Head elevation at least 30°	□Yes □No □Impossible □Prohibited
Rehabilitation	□Yes □No □Not applicable
Deep vein thrombosis	□Yes □Suspicious □No
Anticoagulants	□Applied □Necessary □Not necessary
Inotropic agents	□Can be reduced □Cannot be reduced □No inotropic agent
Administered calories	XX kcal/kg/day
Route of nutrition	□Enteral □Total parenteral □Parenteral
Stress ulcer prophylaxis	□Applied □Necessary □Not necessary
Last defecation	XX days ago
Blood glucose level	□Good □Bad
Correction of serum electrolyte	□Applied □Necessary □Not necessary
Discontinuance of antibiotics	□Possible □Impossible □No antibacterial agent
Unnecessary vascular catheter	□Yes, now extract □No
Unnecessary urinary catheter	□Yes, now extract □No
Today’s goal	(ex) SBT and airway extubation

*RASS* Richmond Agitation Sedation Scale, *SBT* spontaneous breathing trial

**Table 2:** SUP criteria used in the ICU

Major adaptation criteria, at least one of the following	
Coagulopathy (platelets < 50 × 10^3^/mm^3^ and/or APTT < 50% and/or PT-INR ≥ 1.5)	
Mechanical ventilation for more than 48 h	
A history of UGIB within 1 year	
Glasgow Coma Scale ≤ 10	
Thermal injuries to > 35% of their body surface area	
Post partial hepatectomy	
Multiple trauma (Injury Severity Score ≥ 16) [10]	
Transplantation patients	
Hepatic failure	
Spinal cord injury	
Minor adaptation criteria, at least two of the following	
Sepsis	
ICU stay for more than 7 days	
Occult bleeding lasting 6 days or more	
Corticosteroids (> 250 mg/day of hydrocortisone or the equivalent)	
Exclusion criteria: SUP is not required if one of the following	
Enteral nutrition has been already administered	
Early exit from the ICU is expected	

*APTT* activity of activated partial thromboplastin time, *PT-INR* prothrombin time international normalized ratio

Every morning, the medical checklist was reviewed by the ICU physicians and nurses to share the treatment policy and to not forget necessary routine medical practices, such as the SUP. If necessary, the protocols or criteria for the checklist item were referenced (for example, sedation protocol, enteral nutrition protocol, and SUP criteria), and the ICU physicians made a clinical decision about those. Patients who had one of the major adaptation criteria or at least two of the minor adaptation criteria (Table 2) were administered SUP medications if they did not have any exclusion criteria. However, some patients who were considered to be at a higher risk for UGIB could be administered SUP medications at the direction of the ICU attending physician even if they were included in the SUP exclusion criteria.

### Pre- and postinterventional groups

Adult patients (≥ 18 years) admitted to the ICU were classified into the preintervention group (from September to December 21, 2014) and the postintervention group (from December 22, 2014, to April 2015). Readmission to the ICU within 48 h was considered to be consecutive for ICU management. Patients admitted to the ICU for gastrectomy, esophagectomy, pancreatoduodenectomy, and gastrointestinal bleeding were excluded. Patients who did not receive SUP although they were admitted to the ICU in the preintervention period were excluded. As for the postintervention group, patients who received SUP although they did not meet the SUP major or minor criteria were excluded. Patient data before and after the interventional day were collected until the required sample size was fulfilled.

### Outcomes and patient demographics

The primary outcome was the incidence of UGIB defined as one of the following two conditions. First, an ICU physician made a diagnosis of UGIB based on the findings of incessant bloody drainage through a nasogastric tube (NGT) and/or melena. Second, anemia proceeded (the hemoglobin level decreased more than 1 g/dL) within 2 days after there were intermittent signs of bloody drainage through a NGT and/or melena. The NGTs were kept open. The nurses checked the character of the drainage by suctioning the NGTs every 2 to 4 h when administering nutrition and/or medications. The secondary outcome was the proportion of administered SUP medications and the incidence of HAP and CDI. SUP was defined as intravenous or oral H2Bs and/or PPIs. These drugs were considered to be administered if they were administered even once. For example, in a case that we discontinued SUP for some patients on SUP (in case enteral nutrition was started for patients with SUP), these patients were considered to have received a SUP if they were administered a SUP even once. If SUP drugs were changed (from H2B or PPI to another) for any reasons during the ICU stay, both drugs were considered to be administered. HAP was defined if antibiotics were administered against suspected pneumonia at more than 48 h after hospitalization. The definition for CDI included the following findings: presence of diarrhea, positive stool culture result, and positive finding in enzyme immunoassay testing for *Clostridioides difficile* toxin A or B [14].

We also investigated clinically significant UGIB (CSUGIB), which is defined as a UGIB that made patients require at least one of the following: red blood cell transfusion, surgical or endoscopic treatments, and vasopressor agents due to aggravation of the vital signs.

The following patient characteristics were recorded: age, sex, Acute Physiology and Chronic Health Evaluation (APACHE) II score [15], Sequential Organ Failure Assessment (SOFA) score [16] at entry, Glasgow Coma Scale (max score or the ICU exit day’s score if the patient had new central nervous system disorder during ICU stay), length of stay in the ICU, 30-day mortality, admission classification (postoperative or internal, cardiovascular or other surgery), operation time for postoperative patients, UGIB history within 1 year, sepsis (2 points or more increase in SOFA score due to infection [17]), septic shock (sepsis and serum lactate level > 2 mmol/L and necessity of volume resuscitation and vasopressor agents [17]), hepatectomy, hepatic failure (prothrombin time international normalized ratio (PT-INR) ≥ 1.5 resulting from acute liver injury, and/or Child-Pugh classification C cirrhosis), multiple trauma (Injury Severity Score [10] ≥ 16), coagulopathy (platelets < 50 × 10^3^/mm^3^ and/or activity of activated partial thromboplastin time (APTT) < 50% and/or PT-INR ≥ 1.5), thermal injuries (> 35% of their body surface area), organ transplantation, spinal cord injury, duration of mechanical ventilation, blood purification therapy (chronic or intermittent renal replacement therapy and/or plasma exchange), percutaneous cardiopulmonary support, enteral feeding, administration of corticosteroids (> 250 mg/day of hydrocortisone or equivalent), aspirin, warfarin, heparin, and other anticoagulants and/or antiplatelet drugs.

### Statistical analysis

The chi-squared test was utilized to compare the proportions of categorical variables, and the Mann-Whitney *U* test was applied to compare the continuous variables and ordinal variables. The test results for continuous and ordinal variables are reported as the median and interquartile range (IQR) based on the normality of data distribution. All statistical analyses were performed using EZR (Saitama Medical Center, Jichi Medical University, Saitama, Japan), which is a graphical user interface for R (The R Foundation for Statistical Computing, Vienna, Austria, version 3. 3. 1) [18]. For the noninferiority test, one-tailed test was applied while two-tailed tests were performed for other tests. *p* values < 0.05 were considered statistically significant.

### Sample size calculation

Previous studies reported that the incidence of UGIB without SUP in critically ill adults was 5 to 25% [1–3]. From the average value of 15%, it was considered that there was a change range of 10%; therefore, in this study, the noninferiority margin was set at 10%. We received advice from our statistical experts regarding this point. In a preliminary survey at our ICU, we found that the incidence of UGIB in critically ill adults was 4%. If the primary outcome of the preintervention and postintervention groups was 4%, the calculated sample size of every group was 48 (*α* = 0.05, power = 0.8, one-sided test).

## Results

**Fig. 1:**
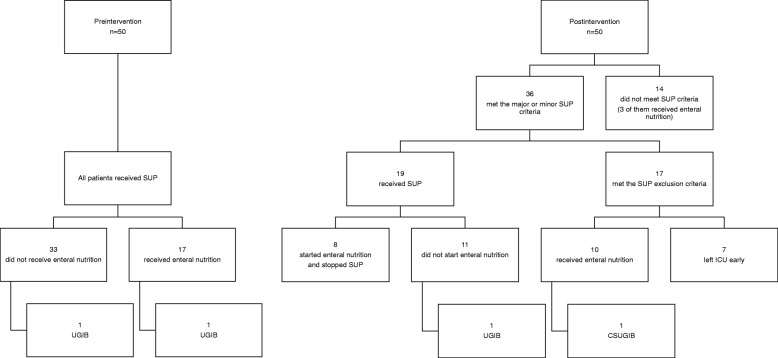
Flowchart of pre- and postintervention groups. SUP, stress ulcer prophylaxis; UGIB, upper gastrointestinal bleeding; CSUGIB, clinically significant UGIB

In total, 100 patients in the pre- (*n* = 50) and postintervention (*n* = 50) groups were analyzed. No patient was classified in duplicate into the pre- and postintervention groups. NGTs were placed in all patients to administer enteral nutrition and medications. As shown in Fig. 1, in the preintervention group, all patients received SUP and 2 patients had UGIB. In the postintervention group, 36 patients were classified into the major or minor SUP criteria. Seventeen of them did not receive SUP because they were included in the SUP exclusion criteria: 10 patients received enteral nutrition and 7 patients were discharged from the ICU after 2 days. Of the 36 patients who were classified into the major or minor SUP criteria, 19 received SUP, 8 started to receive enteral nutrition after the administration of SUP medications, and 1 of the remaining 11 patients who received SUP without enteral nutrition had UGIB. Moreover, one of the patients who were not administered SUP medications because they were already started with enteral nutrition had CSUGIB.

### Patient demographics

No significant differences were found in the patient demographics between the pre- and postintervention groups (Table 3).

**Table 3:** Patient demographics

	Preintervention, *n* = 50	Postintervention, *n* = 50	*p*
Age, years, median (IQR)	67 (60–75)	71 (60–77)	0.54
Sex, male, *n* (%)	33 (66)	25 (50)	0.16
APACHE II score, median (IQR)	15 (10–18)	17 (10–23)	0.16
SOFA score at entry, median (IQR)	4 (2–6)	5 (2–8)	0.46
Glasgow Coma Scale ≤ 10, *n* (%)	6 (12)	8 (16)	0.77
Length of stay in ICU, days, median (IQR)	3 (2–6)	3 (2–9)	0.41
ICU stay for more than 7 days, *n* (%)	12 (24)	17 (34)	0.38
30-day mortality, *n* (%)	7 (14)	4 (8)	0.52
Classification			
Postoperative, cardiovascular, *n* (%)	2 (4)	2 (4)	1.00
Postoperative, others, *n* (%)	32 (64)	25 (50)	0.23
Internal, *n* (%)	16 (32)	23 (46)	0.22
Operation time, min, median (IQR)	449 (198–668)	435 (165–599)	0.59
UGIB history within 1 year, *n* (%)	1 (2)	4 (8)	0.36
Sepsis, *n* (%)	18 (36)	28 (56)	0.07
Septic shock, *n* (%)	6 (12)	11 (22)	0.29
Hepatectomy, *n* (%)	1 (2)	3 (6)	0.61
Hepatic failure, *n* (%)	1 (2)	3 (6)	0.61
Multiple trauma, *n* (%)	0 (0)	2 (4)	0.48
Coagulopathy, *n* (%)	20 (40)	29 (58)	0.11
Mechanical ventilation, hours, median (IQR)	10 (0–59)	18 (3–123)	0.13
Mechanical ventilation for more than 48 h, *n* (%)	14 (28)	19 (38)	0.40
Blood purification therapy, *n* (%)	3 (6)	9 (18)	0.12
Enteral feeding, *n* (%)	17 (34)	21 (42)	0.54
Steroid, *n* (%)	5 (10)	8 (16)	0.55
Aspirin, *n* (%)	3 (6)	3 (6)	1.00
Warfarin, *n* (%)	2 (4)	2 (4)	1.00
Heparin, *n* (%)	8 (16)	14 (28)	0.23
Other anticoagulants or antiplatelets, *n* (%)	5 (10)	11 (22)	0.17

Other anticoagulants or antiplatelet drugs contained low-molecular-weight heparin, danaparoid sodium, nafamostat mesylate, direct thrombin inhibitor, ozagrel sodium, clopidogrel sulfate, and cilostazol

*IQR* interquartile range, *APACHE II* Acute Physiology and Chronic Health Evaluation II, *SOFA* Sequential Organ Failure Assessment, *UGIB* upper gastrointestinal bleeding

### Incidence of UGIB

**Fig. 2:**
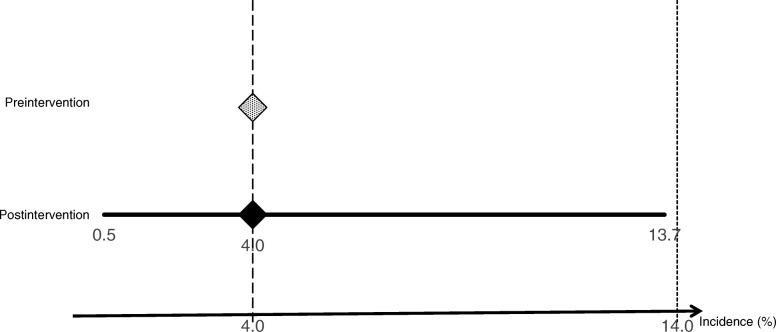
The incidence of UGIB in pre- and postintervention groups. That of preintervention group was 4%. The noninferiority margin was set at 10%; therefore, 4% + 10% = 14% was the predefined line. The incidence of UGIB in postintervention group was 4% [95% CI, 0.5–13.7%]. This upper limit of 95% CI (13.7%) was lower than the predefined line (14%).UGIB, upper gastrointestinal bleeding; CI, confidence interval

No significant difference was found regarding the incidence of UGIB between the groups (Table 4). As previously described, the noninferiority margin for the incidence of UGIB was 10%. The upper limit of 95% confidence interval (CI) of the incidence of UGIB for the postintervention group (13.7%) was lower than the predefined line (4% + 10% = 14%) (Fig. 2). Additionally, no significant difference was found regarding CSUGIB (Table 4).

**Table 4:** Incidence of UGIB and CSUGIB

	Preintervention, *n* = 50	Postintervention, *n* = 50	*p*
UGIB, *n*, % (95% CI)	2, 4% (0.5–13.7%)	2, 4% (0.5–13.7%)	1.00
CSUGIB, *n* (%)	0 (0)	1 (2)	1.00

*UGIB* upper gastrointestinal bleeding, *CSUGIB* clinically significant UGIB, *CI* confidence interval

### Proportion of patients administered with SUP and incidence of HAP and CDI

The proportion of the patients who received a SUP was significantly decreased in the postintervention group compared with that in the preintervention group (Table 5). In preintervention group, PPI was administered to some patients instead of H2B due to renal failure. Additionally, SUP drugs were changed from H2B to PPI in some patients when renal failure was newly diagnosed during their ICU stay. In these cases, the patients in the preintervention group were considered to be administered both H2B and PPI. No significant difference was found regarding the incidence of HAP and CDI (Table 5).

**Table 5:** Proportion of the patients who received SUP and incidence of HAP and CDI

	Preintervention, *n* = 50	Postintervention, *n* = 50	*p*
SUP, *n* (%)	50 (100)	19 (38)	<0.001
H2B, *n* (%)	42* (84*)	7 (14)	
PPI, *n* (%)	15* (30*)	12 (24)	
HAP, *n* (%)	7 (14)	3 (6)	0.32
CDI, *n* (%)	0 (0)	0 (0)	1.00

*SUP* stress ulcer prophylaxis, *H2B* histamine type 2 receptor blocker, *PPI* proton pump inhibitor, *HAP* hospital-acquired pneumonia, *CDI Clostridioides difficile* infection

*Some patients in the preintervention group were administered both H2B and PPI; thus, the total was greater than 100%

## Discussion

To our knowledge, this is the first study to examine whether UGIB increased if SUP was reduced from all to limited cases using a checklist and SUP criteria in the intensive care. Our study showed that the applied interventions could reduce administration of unnecessary SUP and did not increase the incidence of UGIB in critically ill adults, which might have occurred because the SUP criteria facilitated the selection of a high-risk population with stress ulcers, and we did not forget to administer a SUP because of the checklist.

Previous reports on checklists have shown that checklists facilitate ICU medical professionals to administer SUP appropriately and improve compliance. However, no study has reported the benefits of using a medical checklist on the incidence of UGIB with the reduction of unnecessary SUP.

Appropriate selection of a high-risk patient population with stress ulcers is vital to maximize the benefits of SUP with minimum adverse effects. Although it is reasonable that not all patients in the ICU need to receive a SUP, no consensus exists concerning modern intensive care. Presently, a randomized controlled trial comparing a SUP and placebo cannot be carried out due to ethical reasons. Thus, our research can give more insight into this topic. However, it should be noted that one patient, for whom we decided a SUP was unnecessary because enteral nutrition was already started, had CSUGIB. CSUGIB was reported to be associated with higher mortality [19]; therefore, we must reevaluate our work. However, it is unclear whether administration of a SUP to that patient could have prevented UGIB [20]. Moreover, the incidence of CSUGIB was too low to investigate; therefore, further investigations are needed. In detail, although this patient was admitted to the ICU owing to heart and respiratory failure, he had undergone distal gastrectomy two months before this admission. When he entered the ICU, the surgical wound in the digestive tract was thought to have been healed and enteral nutrition was started. However, bloody drainage through the NGT and melena were found; therefore, enteral nutrition was stopped and PPI was administered. Blood transfusions were performed to treat anemia. His condition was improved, and he was discharged from the ICU after 2 weeks.

There were four patients who had UGIB (as shown previously, one of them had CSUGIB) in this study (Fig. 1). One of them in the preintervention group was a patient with septic shock during treatment of hematological malignancy. He had multiple major and minor risk factors for UGIB (coagulopathy, mechanical ventilation for more than 48 h, Glasgow Coma Scale ≤ 10, sepsis, and ICU stay for more than 7 days) (Table 2). Although he was administered both a SUP medication and enteral nutrition, he had UGIB. The cause of UGIB is unknown because gastrointestinal endoscopy was not performed. He died of multiple organ failure associated with septic shock 22 days after entering the ICU. Another patient with UGIB in the preintervention group was admitted to the ICU after an endovascular aneurysm repair for rupture of abdominal aortic aneurysm. Though he also received SUP, he had UGIB. During the endovascular treatment, the radiologist had concerns about transient intestinal ischemia, which was considered to be the cause of UGIB. The patient did not receive enteral nutrition. His condition was improved in 3 days, and he was discharged from the ICU. A patient with UGIB (not CSUGIB) in the postintervention group was admitted to the ICU owing to edema of the airway associated with systemic capillary leak syndrome. She had received SUP, while enteral nutrition had not been started. She fell in cardiopulmonary arrest in the ICU owing to airway obstruction caused by rapid airway edema. After 11 min of cardiopulmonary resuscitation, she was resuscitated. On the day after the resuscitation, bloody drainage in the NGT was found. The cause was thought to be ischemia of the gastrointestinal mucosa owing to cardiopulmonary arrest. Her brain was seriously damaged by cardiopulmonary arrest. A tracheotomy was applied, and she was moved to the general ward while continuing to receive SUP.

This type of intervention might decrease medical costs [21]. Moreover, less opportunity for SUP medication should have resulted reductions of adverse effects, risk of medical errors, and time and effort of nurses. On the other hand, only a few minutes per patient during the morning round would be needed. The incidence of UGIB, length of stay in ICU, and 30-day mortality were not increased; therefore, the total cost of medical care would also not be increased. Therefore, the concept of this intervention can be adapted to any facility.

It was already mentioned that these intervention methods will help us to homogenize the quality of medical care, allowing us to maintain the quality of medical care regardless of facility and staff. Importantly, the methods should be simple and easy to execute, leading to reliability and validity. Additionally, the methods may increase medical professionals’ knowledge about UGIB and SUP. For example, although a computing system that automatically determines the need for a SUP may be useful to homogenize the quality of medical care, it will not increase our knowledge about SUP. It is important for medical professionals to have updated knowledge about SUP.

No significant differences were found in the length of stay in the ICU and 30-day mortality. First, a previous study reported that administration of a SUP in critically ill adults did not improve all-cause mortality [3]. H2Bs and PPIs as SUP have advantages and disadvantages: a decrease in the incidence of UGIB but an increase in HAP and CDI. Although no significant differences were found in the incidence of HAP and CDI, this study was not designed to detect these events. In theory, the risk of these side effects is considered to be reduced because we stopped unnecessary medication. Further research on a larger sample size is needed.

About 4 years have passed since the end of the data collection period, but our checklist and SUP criteria are still in use. The proportion of administered SUP is about 40% now. However, even after the start of enteral nutrition, there seems to be a tendency to continue SUP for patients with higher risk for UGIB.

This study possessed several limitations. First, the reported incidence of UGIB included a wide variation in the results, and the standard treatments in intensive care units may have changed over the past few decades. The quality of intensive care has steadily improved; therefore, the real incidence of UGIB might be lower than we expected. Second, this was a retrospective study; therefore, it was not necessary to detect all UGIB cases precisely. Third, all of the possible confounders were not collected. Fourth, an issue of misclassification may exist in this study. Although we discontinued SUP for some patients with SUP (for example, if enteral nutrition was started for patients with SUP), these patients were considered to have received a SUP if they were administered a SUP even once. Finally, the before and after study may have not captured other modifications in practice over the observed time period. The short timeline (8 months) reduces the risk that some undetected factor changed during the period. We have not evaluated the reliability and validity of the checklist and SUP criteria. Generalization to different facilities and patient populations has not been investigated.

## Conclusions

Utilizing a checklist and SUP criteria reduced unnecessary administration of SUP medications. Regarding the incidence of UGIB in critically ill adults, no significant difference was found between the group that was administered SUP medications on a daily basis and the group restricted to being administered a SUP medication utilizing a checklist and the SUP criteria. In future research, a prospective controlled trial needs to be conducted to evaluate the impact of the proposed method on reducing both the unnecessary usage of SUP medicines and gastrointestinal bleeding.

## Data Availability

The datasets generated and analyzed during the current study are not publicly available. An extract can be available from the corresponding author on reasonable request.

## Abbreviations

APACHE: Acute Physiology and Chronic Health Evaluation
APTT: Activity of activated partial thromboplastin time
CDI: *Clostridioides difficile* infection
CI: Confidence interval
CSUGIB: Clinically significant upper gastrointestinal bleeding
H2B: Histamine type 2 receptor blocker
HAP: Hospital-acquired pneumonia
IQR: Interquartile range
NGT: Nasogastric tube
PPI: Proton pump inhibitor
PT-INR: Prothrombin time international normalized ratio
RASS: Richmond Agitation Sedation Scale
SBT: Spontaneous breathing trial
SOFA: Sequential Organ Failure Assessment
SUP: Stress ulcer prophylaxis
UGIB: Upper gastrointestinal bleeding
